# Inferring predator–prey interaction in the subterranean environment: a case study from Dinaric caves

**DOI:** 10.1038/s41598-021-01249-8

**Published:** 2021-11-04

**Authors:** Ester Premate, Maja Zagmajster, Cene Fišer

**Affiliations:** SubBio Lab, Department of Biology, Biotechnical Faculty, University of Ljubljana, Ljubljana, Slovenia

**Keywords:** Biodiversity, Freshwater ecology, Coevolution

## Abstract

Predator–prey interactions are among the most important biotic interactions shaping ecological communities and driving the evolution of defensive traits. These interactions and their effects on species received little attention in extreme and remote environments, where possibilities for direct observations and experimental manipulation of the animals are limited. In this paper, we study such type of environment, namely caves of the Dinarides (Europe), combining spatial and phylogenetic methods. We focused on several species of *Niphargus* amphipods living in phreatic lakes, as some of them use the dorsal spines as putative morphological defensive traits. We predicted that these spines represent a defense strategy against the olm (*Proteus anguinus*), a top predator species in the subterranean waters. We tested for spatial overlap of the olm and *Niphargus* species and showed that spined species live in closer proximity to and co-occur more frequently with the olm than non-spined species. Modeling of the evolution of the spines onto *Niphargus* phylogeny implies coevolution of this trait in the presence of olm. We conclude that these spines likely evolved as defensive traits in a predator–prey arms race. Combining multiple analyses, we provide an example for a methodological framework to assess predator–prey interactions when in-situ or laboratory observations are not possible.

## Introduction

Predator–prey interactions are among the most important biotic interactions which control community dynamics^1–3^, not only through their lethal outcome for the prey but also through a range of other effects associated with the presence of predators^4–6^. The threat of predation itself can cause the evolution of different defense mechanisms in prey, which either reduce the probability of predators to encounter and attack the prey or the probability to successfully consume it^7,8^. Among others, these mechanisms include habitat shifts, changes in life history, activity levels, and variation in morphological features^9^. Morphological defensive traits, such as particular body shape, spines, or armor, are especially important in the last stage of the predation process and hinder successful catch or consummation of prey^7,10^.

Defensive traits in prey evolve under a constant or only occasional predation pressure and represent a trade-off between fitness costs and successful defense against predators^9^. Permanent defensive traits are more likely to evolve when predators are permanently present, while the occasional presence of predators yields evolution of inducible defense mechanisms^10,11^.

Predator–prey interactions have been extensively studied and the effects of predators on prey are well-documented in many taxa from different communities^1,11,12^. However, these interactions have received little attention so far in communities from extreme and hardly accessible environments, like subterranean habitats or deep sea. The reasons are mainly technical. Rigorous testing of antipredation mechanisms requires experimental manipulation of predator and prey. The experiments on species from remote and extreme environments are hampered given that predator and prey species are hard to collect and even harder to rear and manipulate. The development of comparative and spatial statistical methods may to some extent alleviate these issues and allows indirect assessment of the function of putative defensive traits.

In this study, we explored an alternative approach for the assessment of the predator–prey interactions using a combination of spatial and phylogenetic methods applied to subterranean species from the Dinarides (Western Balkan Peninsula, Southern Europe) as a study system. Dinarides are the home to the largest subterranean amphibian in the world, the olm (*Proteus anguinus* Laurenti, 1768). Its natural range extends from Italy on the northwest to Bosnia and Herzegovina and Montenegro on the southeast^13,14^. It is the largest predator in subterranean habitats, where it represents a constant predation threat to the invertebrates it lives with^15–17^. Here, we explored its relationship with amphipod crustaceans of the genus *Niphargus* Schiödte, 1849, which present the most common subterranean macrocrustaceans in the subterranean waters of the olm range and therefore expected to present an important part of olm’s diet.

**Figure 1:**
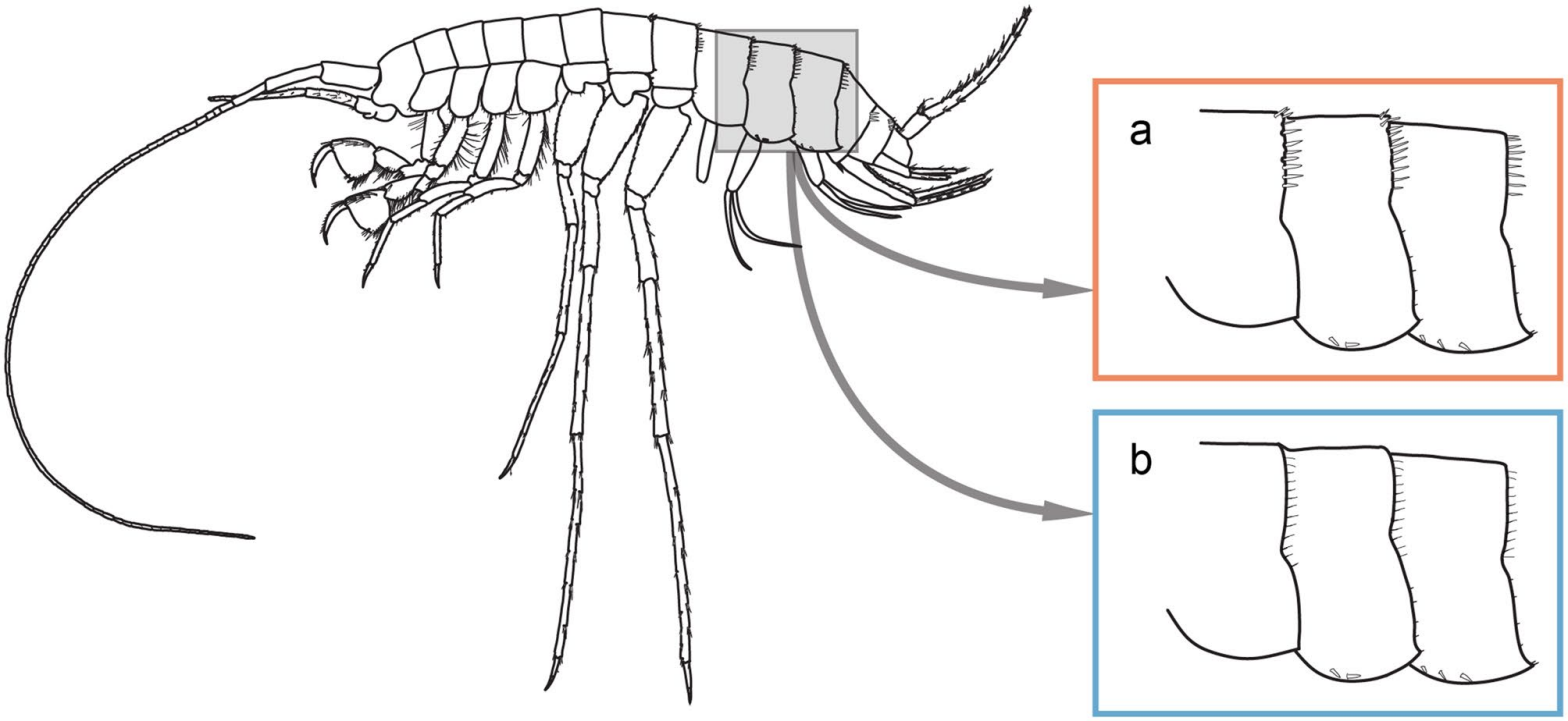
A representative of the lake ecomorph (*Niphargus croaticus*; modified from the previous publication^20^). (**a**) A close-up of the pleon in spined lake *Niphargus* species, with a series of spines along the edge of the segments. (**b**) The same part of the pleon as in non-spined lake *Niphargus* species, with soft and thin setae along the edge of the segments.

*Niphargus* species live in all types of subterranean aquatic habitats^18^. Of special interest to this work are species living in cave lakes, as this is where they frequently co-occur with the olm and where possibilities for escape (e. g. drifting) are limited. *Niphargus* species associated with the cave lakes have a distinct morphology^18^: they are large, stout, and long-legged, and many of them have characteristic dorsal spines on pleon (hereafter referred to as dorsal spines; Fig. 1a). These species were initially attributed to their own subgenus *Orniphargus,* as dorsal spines were considered a synapomorphy of the subgenus and a constituent part of its diagnosis^19^. Subsequent phylogenetic studies revealed that the “subgenus” is polyphyletic and that lake ecomorphs evolved several times independently^18,20^. Moreover, a closer look at all large *Niphargus* species inhabiting subterranean lakes discovered also non-spined species (Fig. 1b). As it was known that some freshwater amphipod genera may evolve defensive dorsal spines or carinas^21,22^, this paved the way for the hypothesis that the dorsal spines are an antipredation mechanism.

To address this hypothesis, we developed three consecutive predictions tested using spatial and phylogenetic comparative methods. First, we predicted that spined lake ecomorphs of *Niphargus* will occur only in the Dinarides, the natural distribution area of the olm. Second, we predicted that spined and non-spined lake ecomorphs of *Niphargus* within the Dinarides will exhibit a different level of spatial overlap with the areas with confirmed presence of the olm. Here we applied two analytical approaches: by evaluating spatial overlap and frequencies of co-occurrences between predator and prey. We quantified distances between the sites with the confirmed occurrence of the olm and lake *Niphargus*, to test whether these distances differ for spined and non-spined lake *Niphargus* species. In addition, we predicted that species with dorsal spines more frequently co-occur with the olm than species without these structures, by using the probabilistic model of species co-occurrence^23^. In the last step, we analyzed the evolution of dorsal spines using the latest *Niphargus* multilocus phylogeny of 373 species^24^. Specifically, we predicted that these spines coevolved in the presence of the olm and that a simple hypothesis of the olm-independent origin of spines would have a smaller explanatory value to the observed distribution of this trait on a phylogenetic tree.

## Results

### Spatial distribution of lake *Niphargus* species and their co-occurrence with the olm

**Figure 2:**
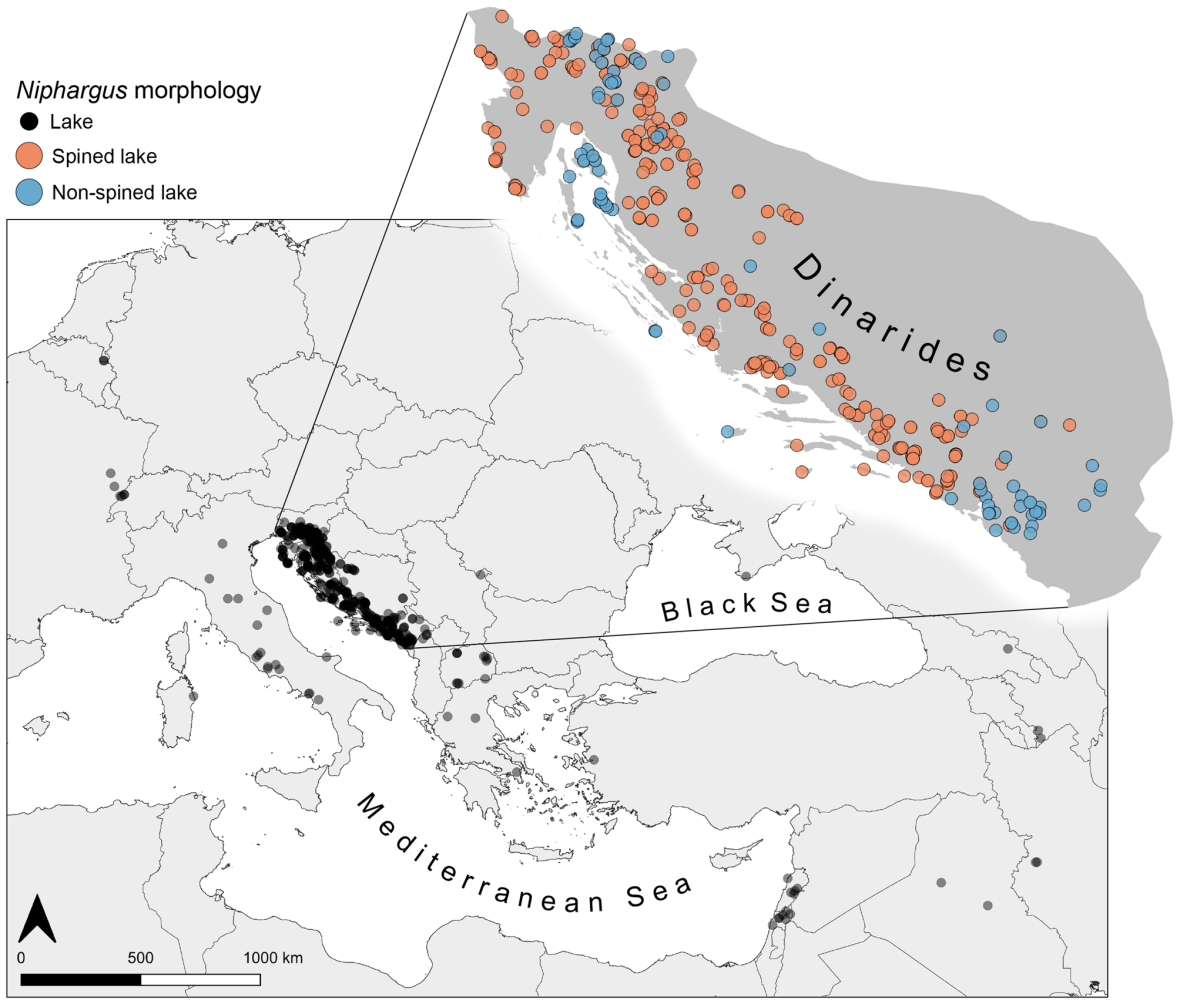
Distribution of all lake *Niphargus* species (black dots) and the detailed distribution of spined and non-spined lake species in the Dinarides. Spined lake species are present only in the depicted area of the Dinarides. Map made with QGIS version 3.10.12.

Mapping all known *Niphargus* localities available in our database^25^ showed that the species of lake ecomorph are distributed from Belgium in the Northwest to Iran in the Southeast, with most occurrences in the Dinarides (Fig. 2). The spined lake ecomorph occurs only in the Dinarides, while non-spined lake ecomorphs can be found across the entire *Niphargus* range (Fig. 2). Within the Dinarides, the non-spined lake species are predominantly distributed in the northwestern and southeastern parts, as well as on the northern Adriatic islands (Fig. 2).

**Figure 3:**
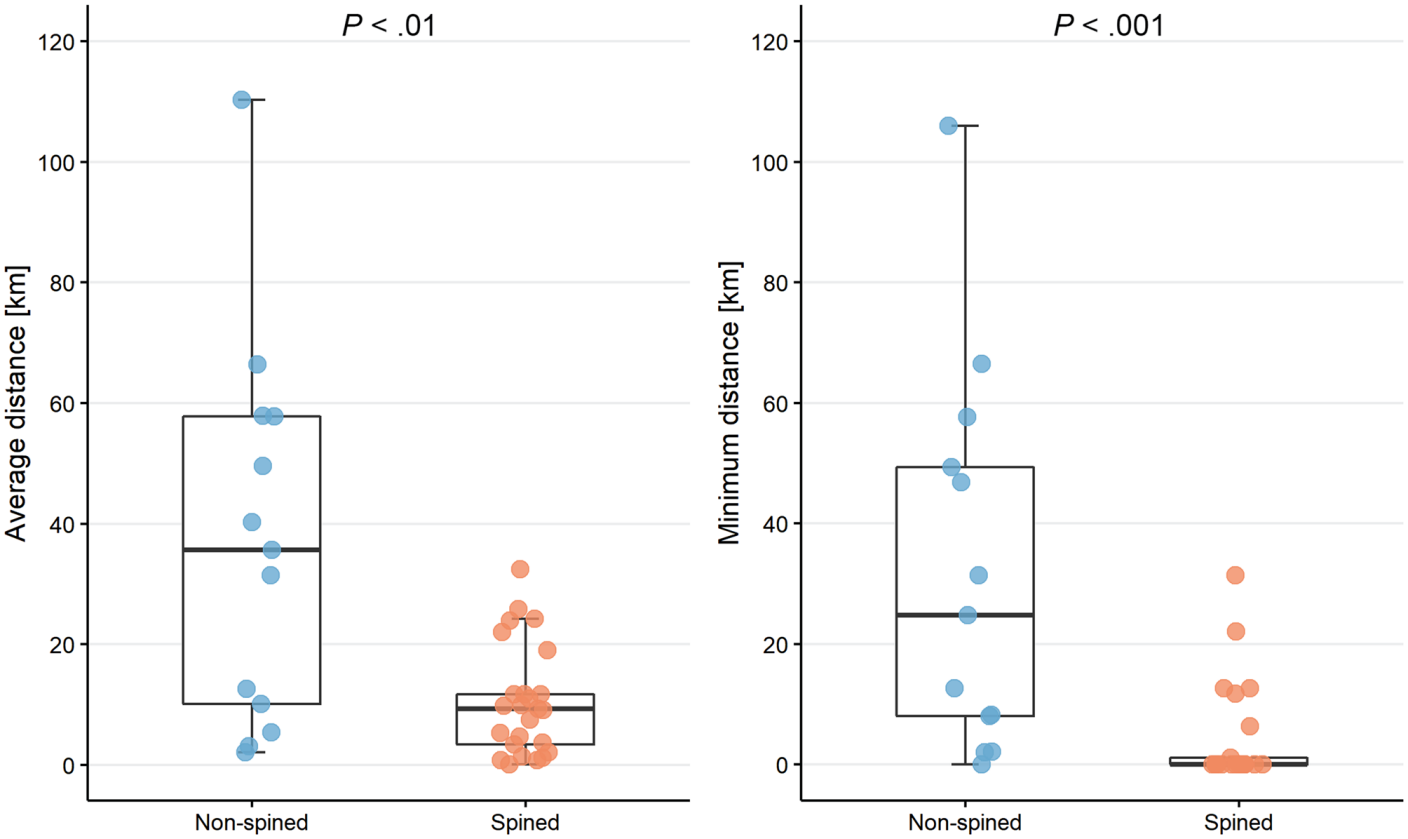
Average (left) and minimum (right) distances of lake *Niphargus* localities to the nearest olm locality by species. Differences between non-spined (n = 13) and spined (n = 38) lake species were assessed with the Kruskal–Wallis rank sum test.

In the next step, we analyzed the distributions of lake *Niphargus* species in relation to the olm within the Dinarides only (Fig. 2, Tables 1 and 2). We calculated Euclidean distances from each lake *Niphargus* locality to the nearest olm locality. Spined lake *Niphargus* species had significantly lower average distances to the nearest olm locality than non-spined lake species (Kruskal–Wallis rank sum test, *P* < 0.01; Fig. 3). Similarly, the minimum distance to the nearest olm locality was significantly lower in spined compared to non-spined lake species (Kruskal–Wallis rank sum test, *P* < 0.001; Fig. 3). The majority (72%) of spined lake species at least once occurred at the same locality as the olm. These results indicate that spined lake *Niphargus* species are on average distributed closer to the olm than non-spined lake *Niphargus* species.

**Table 1:** Counts of lake *Niphargus* and olm co-occurrences by localities.

Localities	No.
Olm	451
Lake *Niphargus*	298
**Olm and lake *Niphargus***	58
Olm and spined *Niphargus*	51
Olm and non-spined *Niphargus*	1
Olm and both morphs *Niphargus*	6

Only co-occurrences in the exact same cave or spring are included.

**Table 2:** Counts of lake *Niphargus* and olm co-occurrences by *Niphargus* species. Only co-occurrences in the exact same cave or spring are included.

Species	No.
**Lake *Niphargus***	38
Spined *Niphargus*	25
Non-spined *Niphargus*	13
Olm and spined *Niphargus*	18
Olm and non-spined *Niphargus*	1
Single co-occurrence	4
More co-occurrences	15

We further assessed the co-occurrence of lake *Niphargus* and the olm by summarizing the presence of both at the same localities. The co-occurrences counted by localities and by species are summarized in Tables 1 and 2.

**Figure 4:**
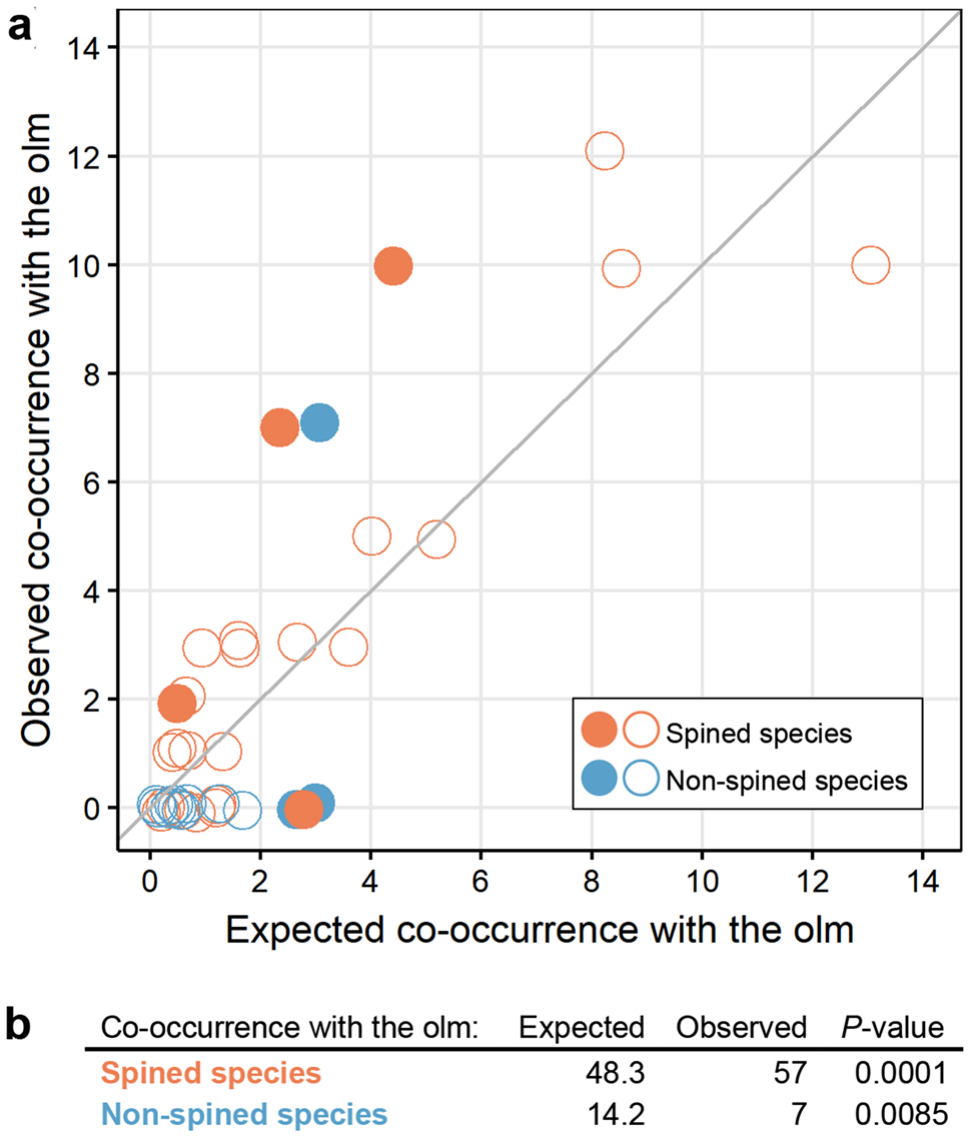
Results of probabilistic models of Dinaric lake *Niphargus* and olm co-occurrence. (**a**) Expected and observed co-occurrences of all lake *Niphargus* species. Full circles represent species with significantly positive (above reference line) or negative (below reference line) co-occurrence patterns with the olm. Empty circles represent species with random co-occurrence patterns with the olm. (**b**) Expected and observed co-occurrence patterns of two morphological groups (spined and non-spined lake). Spined lake species co-occur with the olm more frequently than expected whereas non-spined less frequently than expected.

To statistically quantify pairwise co-occurrence patterns of lake *Niphargus* and the olm, we employed the probabilistic model of species co-occurrence^23^. The model tests the observed against the expected frequencies. Expected frequencies of co-occurrence are assessed from the empirical data. First, we calculated co-occurrence probabilities for each species separately (Fig. 4a). Three spined and one non-spined lake species co-occurred with the olm at a frequency greater than expected. Conversely, two non-spined lake species co-occurred with the olm at a frequency less than expected. Even though nonsignificant, it is noteworthy that most spined and non-spined lake species tended to show more and less frequent co-occurrences with olm, respectively, as compared to theoretical expectation (Fig. 4a).

The non-significant result can be attributed to a relatively limited number of occurrence records per species and thus lower resolution of the method. For this reason, we pooled *Niphargus* species into two morphological groups with respect to the presence of spines and again ran the analysis. This analysis showed that spined lake species co-occurred with the olm at a frequency higher than expected (*P* < 0.001), and non-spined lake species at a frequency less than expected (*P* < 0.01) (Fig. 4b).

### Phylogenetic analysis of *Niphargus’* traits co-evolution

To support the results obtained with spatial analyses, we employed an additional analysis that tested for the correlated evolution of defensive traits in presence of a predator. Using *Niphargus* phylogeny, we tested two evolutionary hypotheses. The first hypothesis stated that the spines developed in correlation with the evolution of the lake ecomorph, through e. g. pleiotropy. The alternative hypothesis stated that the spines developed with the lake ecomorph, but only in species that provenly co-occur with the olm. Both hypotheses were tested against the hypothesis of no correlated evolution. We compared the outputs from different models of spines evolution and evaluated the hypotheses using Log Bayes Factors. The results show strong evidence for correlated evolution both between spines and lake ecomorph and between spines and lake ecomorph co-occurring with the olm (Table 3). The log Bayes Factor was greater in the latter, suggesting that the development of spines in lake *Niphargus* can be better explained with the second hypothesis, i. e. that the spines developed with the lake ecomorph which co-occurs with the olm.

**Table 3:** Results of phylogenetic analysis of *Niphargus*’ traits co-evolution.

Hypothesis	logBF
H1: spines developed with the lake ecomorph	26.76
H2: spines developed with the lake ecomorph, which provenly co-occurs with the olm	71.20

The hypotheses of spines evolution are evaluated using Log Bayes Factor (logBF). Interpretation of Log Bayes Factor values^26^: < 2 weak evidence, > 2 positive evidence, 5–10 strong evidence, > 10 very strong evidence.

## Discussion

A synthesis of the results obtained with different methodological approaches showed that spines found in lake *Niphargus* can be a defensive trait against the olm’s predation*.* The spatial distribution of the spined lake species suggested that the dorsal spines are related to the presence of the olm. Spined lake species on average occurred closer to and shared more localities with the olm than non-spined lake species. The observed co-occurrence of spined lake species and the olm was higher than expected, suggesting that species with this trait are more resilient to predation pressure. Finally, the evolution of spines could have been best explained with the presence of the olm.

Our results are in accord with previous studies on surface freshwater amphipods, where it was shown that spines can be a trait that prolongs the time of ingestion and act as defensive trait^21,22^. However, insights into predator–prey interactions in the subterranean environment have been limited and so far based on field and laboratory observations^15,26,27^ or recognized through food web studies^28–32^. Subterranean vertebrates, such as the olm, are considered apex predators in subterranean communities^33–36^. The defense structures against olm’s predation may be more common than anticipated. There is some evidence that the length of the rostrum of cave shrimp *Troglocaris* may have an antipredator role^15^. Noteworthy, long, and strong cuticular spines with possible defensive function have been found also in some unrelated species of isopod *Monolistra*^37^. These data along with the present study imply that defensive structures might have evolved in three different macro-crustacean genera and indicate that defensive morphological traits are a general response to the olm predation among subterranean crustaceans of the Dinarides.

Although the combination of different methods used in this study has shown congruent and well-supported conclusions, certain aspects call for caution. First, we assumed that the olm and *Niphargus* co-occur only when they are found in the exact same cave, spring, or well, thus discarding the possible groundwater connectivity between localities and co-occurrences in the inaccessible subterranean passages. To compensate for this assumption, we performed additional analyses to assess the co-occurrence of lake *Niphargus* and the olm by adding a buffer of 2 km to point coordinates and evaluated the overlaps between the lake *Niphargus* and the olm polygons. The results were qualitatively similar, and the choice of co-occurrence criterion did not change our conclusions (doi: 10.5281/zenodo.5603235); however, we acknowledge that future phylogeographic analyses of *Niphargus* and olm’s populations may change our perspective on the groundwater connectivity. Second, predator–prey co-occurrence may be temporally variable^4,8^. We assumed that there is no seasonal fluctuation in the occurrence of lake *Niphargus* and the olm within caves. This assumption is likely justified, given the environmental stability of subterranean ecosystems without daily and seasonal environmental fluctuations^38,39^. Third, imperfect detection of species can be an important source of error^40,41^. To some extent, this could be alleviated in the future with the use of environmental DNA^13,42–44^, which would indirectly confirm the presence of a species. Nevertheless, this issue currently remains unsolved as the probabilistic model used in this study does not incorporate the possibility of false species’ presences or absences when estimating the co-occurrence probability^23^.

Our results provide the first evidence for a defensive function of dorsal spines in lake *Niphargus*. Future behavioral tests and experimental predator–prey manipulation would further explore the mechanism behind this defensive trait. However, such experimental work may be difficult to perform in a subterranean model system like ours due to several reasons. First, the olm is a vulnerable^45^ and protected species (listed in Annex II of the Habitats Directive^46^) which can be taken into the laboratory conditions only in a limited amount and under suitable permit. Second, although the olm is still treated as a single species, it has been shown that there is a substantial genetic variation among populations^47,48^ and that it may comprise several cryptic species^48^. Different and independently evolving lineages of olm might have evolved different predation strategies, meaning that the experimental setup should consider the origin of both predator and prey to explain the putative variation of results. This would inflate the number of olm individuals taken from nature. Third, the records in our database^25^ showed that lake *Niphargus* were only rarely found in numbers higher than five specimens per locality, potentially negatively influencing the experiment’s repeatability. If comparative analyses used in this study rejected the hypothesis that spines have a defense function, experimental manipulation of living animals would not be justified. In that perspective, our study can serve as a basis for future laboratory experiments.

Our study provided one of the rare insights into predator–prey interactions in subterranean habitats, and the first using the olm and *Niphargus* species. This raises several interesting questions to be addressed in the future. The first one relates to the only non-spined lake species which regularly co-occurs with the olm, *N. pachytelson*. A detailed examination of the *N. pachytelson*’s localities revealed that other spined lake *Niphargus* species were present in six out of seven co-occurrences with the olm. This suggests that *N. pachytelson* probably evolved other defensive mechanisms against the olm’s predation, which need to be studied. Secondly, by limiting our study to lake *Niphargus*, we excluded *Niphargus* species which are attributed to other cave habitats (e. g. cave streams^18^), yet they may still come into contact with the olm. The stream species are smaller, have shorter appendages, and have more slender body^18^, but no potential morphological defensive traits. These species may employ other defensive strategies, such as e. g. hiding in the substrate or drifting with water currents. However speculative, we hypothesize that environment where species live (limnic-lotic) determines the nature of defensive traits (e.g. morphology versus behavior), a hypothesis that remains to be tested in the future.

Finally, we showed that several alternative, indirect approaches can be used to assess predator–prey interactions and defensive strategies besides conventional *in-situ* observations or laboratory experiments. Such methods carry a potential for even broader usage in the assessment of e. g. food web structure or community dynamics through prey’s functional traits^49,50^ when the functionality of putative defensive traits is validated in laboratory experiments. We argue that the integration of different indirect methods is especially advantageous in hard-to-access environments, where sampling conditions are challenging and focal communities are hardly accessible, providing the groundwater inhabitants as an example.

## Methods

### Data acquisition

We retrieved the data on the distribution of *Niphargus* and the olm from SubBioDB, an internal database comprising the data on subterranean fauna (https://db.subbio.net/), including all known localities and species as of April 2021^25^. We limited the analyses to localities with certain precision of the coordinates (i. e. at least settlement) and excluded the imprecise or unknown localities. We included only lake *Niphargus* species where the species identifications were certain. Spatial datasets could be expanded by assigning more accurate coordinates to some of the localities and by complementing missing species identifications, but such improvements would unlikely affect our conclusions.

For the needs of this study, we categorized lake species into spined and non-spined based on their dorsal armature of pleon segments (Fig. 1). In most cases, this classification was not ambiguous. However, in few species, there are only a few spines, intermixed with thin and flexible setae^20^. In our case, every species in which the spines were present, regardless of their number, was assigned to the “spined” group. Conversely, species that only had setae, were assigned to the “non-spined” group.

Data preparation, manipulation, and visualization were carried out in R version 4.0.3^51^ and RStudio^52^ using packages readxl^53^, xlsx^54^, dplyr^55^, ggplot2^56^, and Ipaper^57^. Spatial analyses were carried out using packages sf^58^, raster^59^, and spatstat^60^, and statistical analyses using rstatix package^61^.

### Spatial analysis of lake *Niphargus’* co-occurrence with the olm

We first visualized the distribution of lake and spined lake *Niphargus* species within the whole range of *Niphargus* species distribution. Later, we limited the spatial analysis to the extent of the Dinarides, as it represents the olm’s natural distribution area and thus the maximum potential area where the olm and *Niphargus* co-occur. We pruned both the olm’s and *Niphargus’* datasets to the extent of the Dinarides with an additional buffer of 3 km to also include island and border localities.

First, we calculated the Euclidean distances of each lake *Niphargus* locality to the nearest olm locality. We further calculated average distances per lake *Niphargus* species and evaluated the differences between spined and non-spined groups using the Kruskal–Wallis rank sum test.

We counted the localities where both the olm and lake *Niphargus’* co-occur, separating spined and non-spined ecomorphs. Separately, we counted the number of *Niphargus* species which co-occur with the olm, again based on their morphology. The co-occurrence was in all cases restricted to the occurrence in the exact same cave or spring.

### Probabilistic model of lake *Niphargus* and olm co-occurrence

We tested pairwise co-occurrence patterns of lake *Niphargus* species and the olm using a probabilistic model of species co-occurrence with hypergeometric distribution within the R package cooccur^62^. Co-occurrence probabilities were calculated on a matrix of all lake *Niphargus* species and localities in the Dinarides. Additionally, we pooled the species in two morphological groups (spined lake *Niphargus,* non-spined lake *Niphargus*) and calculated the co-occurrence probabilities of both groups with the olm. We retrieved the observed and expected co-occurrences of lake *Niphargus* species with the olm and evaluated whether co-occurrences patterns of the defined groups are significantly positive, negative, or random^62^.

### Phylogenetic analysis of *Niphargus*’ traits co-evolution

Dorsal spines in lake *Niphargus* could have evolved in diverse testable scenarios. We proposed two hypotheses which were tested using phylogenetic comparative methods. The simplest first hypothesis stated that the dorsal spines develop with the evolution of the lake ecomorph through e. g. pleiotropy. The alternative hypothesis stated that the dorsal spines developed with the lake ecomorph which provenly co-occurs with the olm.

For the needs of comparative methods, we additionally assembled matrices of three discrete traits data for each *Niphargus* species. The traits included (i) the presence or absence of dorsal spines, (ii) overall species morphology, i.e. whether it can be attributed to the group of *Orniphargus* (the so-called lake ecomorph^18^), and (iii) confirmed co-occurrence with the olm. The latter was based on preliminary spatial analysis. The matrices of traits data comprised only species included in *Niphargus* phylogeny (see below), and not all the species that were present in the distribution dataset used in spatial analyses.

We tested whether spines co-evolve with any of the other two discrete traits using the latest available *Niphargus* multilocus phylogeny of 373 species^24^. We extracted 1,000 random trees out of 10,000 trees from the stationary phase in Bayesian phylogenetic analysis. We used phytools package in R^63^ for phylogenetic trees import and manipulation. Each of the three hypotheses of correlated evolution was tested against the alternative hypothesis of no correlated evolution.

We carried out the analysis in BayesTraits version 3.0.1^64^, run from R using the package btw^65^. First, we tested dependent and independent maximum likelihood (MLH) models of trait evolution for all three hypotheses using the random sample of 100 phylogenetic trees. Based on the output of the ML models, we set the interval of uniform priors used in Markov Chain Monte Carlo (MCMC) analyses. We performed MCMC analysis for both dependent and independent models of trait evolution for all three hypotheses on the sample of 1000 phylogenetic trees, using the following settings: two MCMC chains, run for 10 000 000 iterations, with the burn-in set to 1 000 000 and stepping-stone sampler set to 500 stones, each run for 1000 iterations. The latter was used to estimate the marginal likelihood (MarLH) of both models, further used in the calculations of log Bayes Factors (see^64^ for details). Finally, we evaluated which hypothesis on the evolution of dorsal spines is the most plausible based on log Bayes Factors.

## Data Availability

Data used in this study is available on Zenodo at 10.5281/zenodo.5603098.

## Code availability

R source code is available on Zenodo at 10.5281/zenodo.5603235.
